# Penta­europium dicadmium penta­anti­monide oxide, Eu_5_Cd_2_Sb_5_O

**DOI:** 10.1107/S1600536811000274

**Published:** 2011-01-15

**Authors:** Bayrammurad Saparov, Svilen Bobev

**Affiliations:** a Department of Chemistry and Biochemistry, University of Delaware, Newark, DE 19716, USA

## Abstract

The title compound, Eu_5_Cd_2_Sb_5_O adopts the Ba_5_Cd_2_Sb_5_F-type structure (Pearson symbol *oC*52), which contains nine crystallographically unique sites in the asymmetric unit, all on special positions. One Eu, two Sb, and the Cd atom have site symmetry *m*..; two other Eu, the third Sb and the O atom have site symmetry *m*2*m*; the remaining Eu atom has 2/*m*.. symmetry. Eu atoms fill penta­gonal channels built from corner-sharing CdSb_4_ tetra­hedra. The isolated O atom, *i.e.*, an oxide ion O^2−^, is located in a distorted tetra­hedral cavity formed by four Eu cations.

## Related literature

For related ternary pnictides, see: Xia & Bobev (2007*a* ▶,*b* ▶, 2008*a* ▶,*b* ▶); Saparov *et al.* (2008*a* ▶,*b* ▶, 2010 ▶, 2011 ▶); Park & Kim (2004 ▶). For related anti­monide fluorides and oxides [*A*_5_Cd_2_Sb_5_F (*A* = Sr, Ba, Eu); Ba_5_Cd_2_Sb_5_O_*x*_], see: Saparov & Bobev (2010 ▶). For another related bis­muthide oxide (Ba_2_Cd_2.13_Bi_3_O), see: Xia & Bobev (2010 ▶). For ionic and covalent radii, see: Shannon (1976 ▶); Pauling (1960 ▶).

## Experimental

### 

#### Crystal data

Eu_5_Cd_2_Sb_5_O
*M* *_r_* = 1609.37
Orthorhombic, 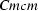

*a* = 4.7088 (5) Å
*b* = 21.965 (2) Å
*c* = 14.5982 (15) Å
*V* = 1509.9 (3) Å^3^
*Z* = 4
Mo *K*α radiation
μ = 31.92 mm^−1^
*T* = 120 K
0.06 × 0.05 × 0.04 mm

#### Data collection

Bruker SMART APEX diffractometer
Absorption correction: multi-scan (*SADABS*; Bruker, 2002 ▶) *T*_min_ = 0.161, *T*_max_ = 0.279
10204 measured reflections
1092 independent reflections
1031 reflections with *I* > 2σ(*I*)
*R*_int_ = 0.043

#### Refinement

*R*[*F* ^2^ > 2σ(*F* ^2^)] = 0.020
*wR*(*F* ^2^) = 0.044
*S* = 1.14
1092 reflections
46 parameters
Δρ_max_ = 1.18 e Å^−3^
Δρ_min_ = −1.16 e Å^−3^

### 

Data collection: *SMART* (Bruker, 2002 ▶); cell refinement: *SAINT* (Bruker, 2002 ▶); data reduction: *SAINT*; program(s) used to solve structure: *SHELXTL* (Sheldrick, 2008 ▶); program(s) used to refine structure: *SHELXTL*; molecular graphics: *XP* in *SHELXTL*; software used to prepare material for publication: *SHELXTL*.

## Supplementary Material

Crystal structure: contains datablocks global, I. DOI: 10.1107/S1600536811000274/mg2112sup1.cif

Structure factors: contains datablocks I. DOI: 10.1107/S1600536811000274/mg2112Isup2.hkl

Additional supplementary materials:  crystallographic information; 3D view; checkCIF report

## Figures and Tables

**Fig. 1.**
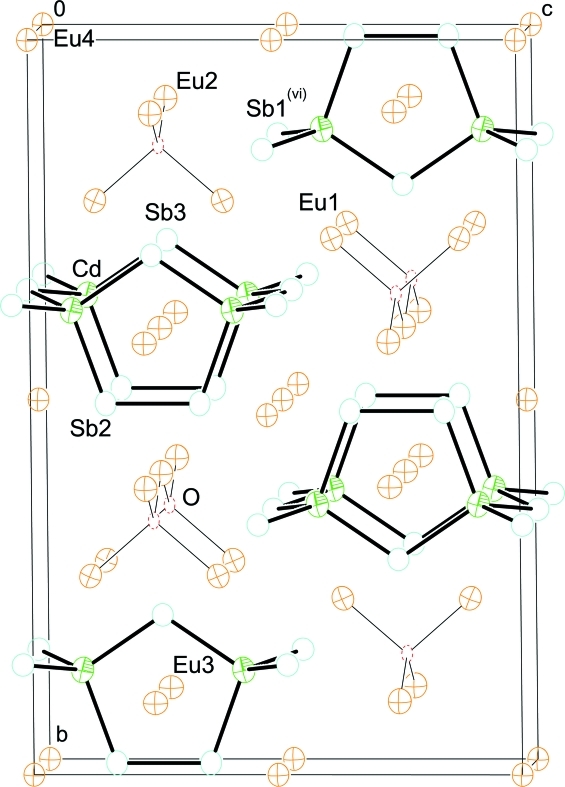
Projection of Eu5Cd2Sb5O approximately along [100]. Displacement ellipsoids are drawn at the 95% probability level. Color key: Eu orange, Cd green, Sb turquoise, O red. Symmetry transformation used to generate the equivalent Sb atom: (vi) x,y,-z+1/2.

## supplementary crystallographic information

### Comment

The title compound, Eu_5_Cd_2_Sb_5_O, is isostructural to Ba_5_Cd_2_Sb_5_F, recently reported (Saparov & Bobev, 2010). The structure contains one-dimensional [Cd_2_Sb_5_]^9-^ polyanions and isolated O^2-^ ions surrounded tetrahedrally by Eu cations (Fig. 1). The polyanions are constructed from two chains of corner-sharing CdSb_4_ tetrahedra connected through Sb3 atoms and Sb2–Sb2 bonds [2.8078 (10) Å]. Similar strong homoatomic Sb–Sb interactions are found in related polyanionic Zn–Sb and Cd–Sb substructures [Ba_3_Cd_2_Sb_4_ (Saparov *et al.*, 2008*a*), Sr_11_Cd_6_Sb_12_ (Park & Kim, 2004; Xia & Bobev, 2008*a*), Eu_11_Cd_6_Sb_12_ and Eu_11_Cd_6_Sb_12_ (Saparov *et al.*, 2008*b*), Ba_2_Cd_2_Sb_3_ (Saparov *et al.*, 2010)]. The Cd–Sb interactions [2.8413 (5) Å to 2.9624 (8) Å] are comparable to the sum of the covalent radii (Pauling, 1960) and to distances found in other structures based on CdSb_4_ tetrahedra [Ba_3_Cd_2_Sb_4_ (Saparov *et al.*, 2008*a*), Ba_21_Cd_4_Sb_18_ (Xia and Bobev, 2008*b*), Eu_11_Cd_6_Sb_12_ (Saparov *et al.*, 2008*b*), *A*_2_CdSb_2_ (Xia & Bobev, 2007*a*; Saparov *et al.*, 2011), Sr_9_Cd_4.49 (1)_Sb_9_ (Xia & Bobev, 2007*b*)].

Band structure calculations highlight the importance of ionic Ba–F interations near the Fermi level to optimize bonding in Ba_5_Cd_2_Sb_5_F, but exact electron balance is achieved in the corresponding oxide Ba_5_Cd_2_Sb_5_O*_x_* only when *x* = 0.5 (Saparov & Bobev, 2010). Whereas the fluoride is free of disorder, the oxide exhibits underoccupancy of the oxygen site, causing positional disorder of the next-nearest Ba atoms, as revealed by elongated atomic displacement parameters on the Ba2 site (modeled as a split position) in Ba_5_Cd_2_Sb_5_O_0.59 (3)_. The Ba2 atoms move away from their equilibrium positions towards the empty space that results when the oxygen site is vacant. Thus, it is surprising that the present structure of Eu_5_Cd_2_Sb_5_O contains a fully occupied oxygen site, because the formula would show a one-electron deficiency, *viz.* (Eu^2+^)_5_(Cd^2+^)_2_(Sb^3-^)_3_(Sb^2-^)_2_(O^2-^). A possible resolution is to propose the occurrence of Eu in both +2 and +3 oxidation states. Because phase-pure samples were unavailable, magnetic measurements could not be performed to verify this proposal. Nevertheless, we note that the Eu1–O distance [2.528 (4) Å] is only 0.4% longer than the Eu1–F distance in Eu_5_Cd_2_Sb_5_F (Saparov & Bobev, 2010); this increase does not scale with the difference between the ionic radii of O^2-^ and F^-^, the former being nearly 5% bigger than the latter (Shannon, 1976). A similar conclusion can be drawn by comparing the Eu2–O [2.634 (3) Å] and Eu2–F [2.635 (3) Å] distances.

### Experimental

The reagents were handled in an argon-filled glove box or under vacuum. All metals were with a stated purity higher than 99.9% (metal basis). They were purchased from Alfa, kept in the glove box, and were used as received.

A mixture of elemental Eu, Cd, Sb, and Pb (flux) in a molar ratio Eu:Cd:Sb:Pb = 2:1:2:10 was loaded in an alumina crucible. To prevent oxidation, the elements were weighed inside the glove box, but the mixture was accidently left in contact with ambient air for *ca.* 20–30 minutes, prior to sealing it under vacuum inside a silica tube. After that, the reaction mixture was put in a box-furnace and heated to 1273 K at a rate of 200 K h^-1^, homogenized at this temperature for 24 h, and then slowly cooled to 823 K at a rate of 5 K h^-1^. After an equilibration step at 823 K for 96 h, the crystals were separated from the Pb flux.

This reaction was aimed at obtaining large single-crystals of Eu_11_Cd_6_Sb_12_ (Saparov *et al.*, 2008*b*), which was indeed the major product. However, alongside the needle crystals of Eu_11_Cd_6_Sb_12_, a small block-shaped crystal was also found. After the X-ray data were collected and the structure was solved, it turned out to be that of Eu_5_Cd_2_Sb_5_O. Other reactions using the same starting materials produced only the intermetallic phase, suggesting that the likely source of oxygen in this particular experiment was an unexpected partial oxidation. Attempts to increase the yield by using Eu_2_O_3_ as a deliberate source of oxygen were not successful and yielded multiple phases. Reactions aimed at obtaining the isostructural Eu_5_Cd_2_Sb_5_F (Saparov & Bobev, 2010) were successful – they were performed using CdF_2_ (Alfa), Eu, Cd, and Sb.

### Refinement

Because the determined unit-cell dimensions and space group suggested isomorphism with Ba_5_Cd_2_Sb_5_F (Saparov & Bobev, 2010), the diffraction data were readily refined using this model. The refinements smoothly converged to low conventional residuals and a flat difference Fourier map. The maximum peak and deepest hole were located 0.86 Å from Eu2 and 0.93 Å from O, respectively.

We note that when oxygen was excluded from the model, a residual peak of about 15 e^-^ Å^-3^, located *ca.* 2.6–2.7 Å from Eu, remained in the difference Fourier map. Unlike the case of Ba_2_Cd_3-__δ_Bi_3_O (Xia & Bobev, 2010), where the residual density lacked the typical oxoanion coordination and was modeled as a partially occupied Cd site, here the tetrahedral coordination by Eu matches very well the bonding requirements of O^2-^. The distances are reasonable and the oxygen site was fully occupied, as verified by freeing the site occupation factor, which led to an occupancy factor of 1.05 (2). In the final refinement cycles, all atoms were refined as fully occupied.

### Crystal data

**Table:**

Eu_5_Cd_2_Sb_5_O	*F*(000) = 2702
*M**_r_* = 1609.37	*D*_x_ = 7.078 Mg m^−^^3^
Orthorhombic, *C**m**c**m*	Mo *K*α radiation, λ = 0.71073 Å
Hall symbol: -C 2c 2	Cell parameters from 1092 reflections
*a* = 4.7088 (5) Å	θ = 1.9–28.3°
*b* = 21.965 (2) Å	µ = 31.92 mm^−^^1^
*c* = 14.5982 (15) Å	*T* = 120 K
*V* = 1509.9 (3) Å^3^	Block, black
*Z* = 4	0.06 × 0.05 × 0.04 mm

### Data collection

**Table:**

Bruker SMART APEX diffractometer	1092 independent reflections
Radiation source: fine-focus sealed tube	1031 reflections with *I* > 2σ(*I*)
graphite	*R*_int_ = 0.043
ω scans	θ_max_ = 28.3°, θ_min_ = 1.9°
Absorption correction: multi-scan (*SADABS*; Bruker, 2002)	*h* = −6→6
*T*_min_ = 0.161, *T*_max_ = 0.279	*k* = −28→28
10204 measured reflections	*l* = −19→19

### Refinement

**Table:**

Refinement on *F*^2^	Primary atom site location: structure-invariant direct methods
Least-squares matrix: full	Secondary atom site location: difference Fourier map
*R*[*F*^2^ > 2σ(*F*^2^)] = 0.020	*w* = 1/[σ^2^(*F*_o_^2^) + (0.0174*P*)^2^ + 9.1021*P*] where *P* = (*F*_o_^2^ + 2*F*_c_^2^)/3
*wR*(*F*^2^) = 0.044	(Δ/σ)_max_ = 0.001
*S* = 1.14	Δρ_max_ = 1.18 e Å^−^^3^
1092 reflections	Δρ_min_ = −1.16 e Å^−^^3^
46 parameters	Extinction correction: *SHELXTL* (Bruker, 2002), Fc^*^=kFc[1+0.001xFc^2^λ^3^/sin(2θ)]^-1/4^
0 restraints	Extinction coefficient: 0.000112 (14)

### Special details

**Table:**

Experimental. Selected in the glove box, crystals were put in a Paratone N oil and cut to the desired dimensions. Chosen crystal was mounted on a tip of a glass fiber and quickly onto the goniometer. The crystal was kept under a cold nitrogen stream to protect from ambient conditions.Data collection is performed with four batch runs at φ = 0.00 ° (600 frames), at φ = 90.00 ° (600 frames), at φ = 180.00 ° (600 frames), and at φ = 270.00 (600 frames). Frame width = 0.30 ° in ω. Data are merged and treated with multi-scan absorption corrections.
Geometry. All e.s.d.'s (except the e.s.d. in the dihedral angle between two l.s. planes) are estimated using the full covariance matrix. The cell e.s.d.'s are taken into account individually in the estimation of e.s.d.'s in distances, angles and torsion angles; correlations between e.s.d.'s in cell parameters are only used when they are defined by crystal symmetry. An approximate (isotropic) treatment of cell e.s.d.'s is used for estimating e.s.d.'s involving l.s. planes.
Refinement. Refinement of *F*^2^ against ALL reflections. The weighted *R*-factor *wR* and goodness of fit *S* are based on *F*^2^, conventional *R*-factors *R* are based on *F*, with *F* set to zero for negative *F*^2^. The threshold expression of *F*^2^ > σ(*F*^2^) is used only for calculating *R*-factors(gt) *etc*. and is not relevant to the choice of reflections for refinement. *R*-factors based on *F*^2^ are statistically about twice as large as those based on *F*, and *R*- factors based on ALL data will be even larger.

### Fractional atomic coordinates and isotropic or equivalent isotropic displacement parameters (Å^2^)

**Table:**

	*x*	*y*	*z*	*U*_iso_*/*U*_eq_	
Eu1	0.0000	0.270509 (17)	0.61940 (3)	0.01436 (11)	
Eu2	0.0000	0.10016 (3)	0.2500	0.02034 (14)	
Eu3	0.0000	0.90247 (2)	0.2500	0.01461 (12)	
Eu4	0.0000	0.0000	0.0000	0.01393 (12)	
Sb1	0.0000	0.14937 (2)	0.02068 (3)	0.01333 (12)	
Sb2	0.0000	0.49492 (2)	0.15383 (3)	0.01439 (12)	
Sb3	0.0000	0.29312 (3)	0.2500	0.01319 (15)	
Cd	0.0000	0.36803 (3)	0.08509 (4)	0.01540 (13)	
O	0.0000	0.6539 (3)	0.2500	0.0058 (12)	

### Atomic displacement parameters (Å^2^)

**Table:**

	*U*^11^	*U*^22^	*U*^33^	*U*^12^	*U*^13^	*U*^23^
Eu1	0.01347 (18)	0.01609 (19)	0.01352 (19)	0.000	0.000	0.00131 (14)
Eu2	0.0283 (3)	0.0179 (3)	0.0148 (3)	0.000	0.000	0.000
Eu3	0.0119 (2)	0.0152 (3)	0.0167 (3)	0.000	0.000	0.000
Eu4	0.0145 (3)	0.0151 (3)	0.0122 (2)	0.000	0.000	−0.00009 (19)
Sb1	0.0121 (2)	0.0142 (2)	0.0136 (2)	0.000	0.000	−0.00025 (18)
Sb2	0.0137 (2)	0.0171 (2)	0.0123 (2)	0.000	0.000	0.00042 (18)
Sb3	0.0123 (3)	0.0146 (3)	0.0127 (3)	0.000	0.000	0.000
Cd	0.0137 (3)	0.0186 (3)	0.0140 (3)	0.000	0.000	−0.0012 (2)
O	0.006 (3)	0.008 (3)	0.003 (3)	0.000	0.000	0.000

### Geometric parameters (Å, °)

**Table:**

Eu1—O^i^	2.528 (4)	Sb1—Eu1^xvi^	3.2738 (5)
Eu1—Sb1^ii^	3.2738 (5)	Sb1—Eu1^xvii^	3.2738 (5)
Eu1—Sb1^iii^	3.2738 (5)	Sb1—Eu1^vi^	3.3559 (7)
Eu1—Sb3^iv^	3.3363 (4)	Sb2—Sb2^vi^	2.8078 (10)
Eu1—Sb3^v^	3.3363 (4)	Sb2—Cd	2.9624 (8)
Eu1—Sb1^vi^	3.3559 (7)	Sb2—Eu4^ix^	3.2556 (4)
Eu2—O^vii^	2.634 (3)	Sb2—Eu4^x^	3.2556 (4)
Eu2—O^viii^	2.634 (3)	Sb2—Eu3^viii^	3.4115 (5)
Eu3—Sb3^ix^	3.3634 (7)	Sb2—Eu3^vii^	3.4115 (5)
Eu3—Sb3^x^	3.3634 (7)	Sb3—Cd	2.9160 (7)
Eu3—Sb2^xi^	3.4115 (5)	Sb3—Cd^vi^	2.9160 (7)
Eu3—Sb2^x^	3.4115 (5)	Sb3—Eu1^xvii^	3.3363 (4)
Eu3—Sb2^ix^	3.4115 (5)	Sb3—Eu1^iv^	3.3363 (4)
Eu3—Sb2^xii^	3.4115 (5)	Sb3—Eu1^v^	3.3363 (4)
Eu3—Cd^ix^	3.4512 (5)	Sb3—Eu1^xvi^	3.3363 (4)
Eu3—Cd^xi^	3.4512 (5)	Sb3—Eu3^vii^	3.3634 (7)
Eu3—Cd^xii^	3.4512 (5)	Sb3—Eu3^viii^	3.3634 (7)
Eu3—Cd^x^	3.4512 (5)	Cd—Sb1^xiii^	2.8413 (5)
Eu4—Sb2^xiii^	3.2556 (4)	Cd—Sb1^xiv^	2.8413 (5)
Eu4—Sb2^vii^	3.2556 (4)	Cd—Eu3^vii^	3.4512 (5)
Eu4—Sb2^xiv^	3.2556 (4)	Cd—Eu3^viii^	3.4512 (5)
Eu4—Sb2^viii^	3.2556 (4)	O—Eu1^i^	2.528 (4)
Eu4—Sb1	3.2947 (6)	O—Eu1^xviii^	2.528 (4)
Eu4—Sb1^xv^	3.2948 (6)	O—Eu2^ix^	2.634 (3)
Sb1—Cd^xiii^	2.8413 (5)	O—Eu2^x^	2.634 (3)
Sb1—Cd^xiv^	2.8413 (5)		
			
O^i^—Eu1—Sb1^ii^	88.79 (8)	Sb2^xiii^—Eu4—Sb2^viii^	87.362 (15)
O^i^—Eu1—Sb1^iii^	88.79 (8)	Sb2^vii^—Eu4—Sb2^viii^	92.638 (15)
Sb1^ii^—Eu1—Sb1^iii^	91.972 (17)	Sb2^xiv^—Eu4—Sb2^viii^	180.00 (2)
O^i^—Eu1—Sb3^iv^	81.04 (8)	Sb2^xiii^—Eu4—Sb1	91.665 (10)
Sb1^ii^—Eu1—Sb3^iv^	88.237 (12)	Sb2^vii^—Eu4—Sb1	88.335 (10)
Sb1^iii^—Eu1—Sb3^iv^	169.821 (18)	Sb2^xiv^—Eu4—Sb1	91.665 (10)
O^i^—Eu1—Sb3^v^	81.04 (8)	Sb2^viii^—Eu4—Sb1	88.335 (10)
Sb1^ii^—Eu1—Sb3^v^	169.821 (18)	Sb2^xiii^—Eu4—Sb1^xv^	88.335 (10)
Sb1^iii^—Eu1—Sb3^v^	88.237 (12)	Sb2^vii^—Eu4—Sb1^xv^	91.665 (10)
Sb3^iv^—Eu1—Sb3^v^	89.772 (15)	Sb2^xiv^—Eu4—Sb1^xv^	88.335 (10)
O^i^—Eu1—Sb1^vi^	168.60 (11)	Sb2^viii^—Eu4—Sb1^xv^	91.665 (10)
Sb1^ii^—Eu1—Sb1^vi^	99.089 (14)	Sb1—Eu4—Sb1^xv^	180.0
Sb1^iii^—Eu1—Sb1^vi^	99.089 (14)	Sb2^xiii^—Eu4—Cd^xiv^	100.934 (11)
Sb3^iv^—Eu1—Sb1^vi^	90.921 (15)	Sb2^vii^—Eu4—Cd^xiv^	79.066 (11)
Sb3^v^—Eu1—Sb1^vi^	90.921 (15)	Sb2^xiv^—Eu4—Cd^xiv^	47.500 (11)
O^i^—Eu1—Cd^vi^	103.28 (11)	Sb2^viii^—Eu4—Cd^xiv^	132.500 (11)
Sb1^ii^—Eu1—Cd^vi^	47.849 (9)	Sb1—Eu4—Cd^xiv^	45.208 (9)
Sb1^iii^—Eu1—Cd^vi^	47.849 (9)	Sb1^xv^—Eu4—Cd^xiv^	134.793 (9)
Sb3^iv^—Eu1—Cd^vi^	135.111 (8)	Sb2^xiii^—Eu4—Cd^viii^	79.066 (11)
Sb3^v^—Eu1—Cd^vi^	135.111 (7)	Sb2^vii^—Eu4—Cd^viii^	100.934 (11)
Sb1^vi^—Eu1—Cd^vi^	88.118 (17)	Sb2^xiv^—Eu4—Cd^viii^	132.500 (11)
O^i^—Eu1—Eu1^xix^	41.06 (11)	Sb2^viii^—Eu4—Cd^viii^	47.500 (11)
Sb1^ii^—Eu1—Eu1^xix^	116.120 (11)	Sb1—Eu4—Cd^viii^	134.792 (9)
Sb1^iii^—Eu1—Eu1^xix^	116.120 (11)	Sb1^xv^—Eu4—Cd^viii^	45.207 (9)
Sb3^iv^—Eu1—Eu1^xix^	55.150 (8)	Cd^xiv^—Eu4—Cd^viii^	180.000 (19)
Sb3^v^—Eu1—Eu1^xix^	55.150 (8)	Sb2^xiii^—Eu4—Cd^xiii^	47.500 (11)
Sb1^vi^—Eu1—Eu1^xix^	127.542 (11)	Sb2^vii^—Eu4—Cd^xiii^	132.500 (11)
Cd^vi^—Eu1—Eu1^xix^	144.340 (11)	Sb2^xiv^—Eu4—Cd^xiii^	100.934 (11)
O^i^—Eu1—Cd^iii^	127.77 (7)	Sb2^viii^—Eu4—Cd^xiii^	79.066 (11)
Sb1^ii^—Eu1—Cd^iii^	143.242 (16)	Sb1—Eu4—Cd^xiii^	45.208 (9)
Sb1^iii^—Eu1—Cd^iii^	85.892 (12)	Sb1^xv^—Eu4—Cd^xiii^	134.793 (9)
Sb3^iv^—Eu1—Cd^iii^	99.991 (16)	Cd^xiv^—Eu4—Cd^xiii^	73.486 (12)
Sb3^v^—Eu1—Cd^iii^	46.924 (12)	Cd^viii^—Eu4—Cd^xiii^	106.514 (12)
Sb1^vi^—Eu1—Cd^iii^	45.530 (10)	Sb2^xiii^—Eu4—Cd^vii^	132.500 (11)
Cd^vi^—Eu1—Cd^iii^	110.630 (13)	Sb2^vii^—Eu4—Cd^vii^	47.500 (11)
Eu1^xix^—Eu1—Cd^iii^	97.418 (10)	Sb2^xiv^—Eu4—Cd^vii^	79.066 (11)
O^i^—Eu1—Cd^ii^	127.77 (7)	Sb2^viii^—Eu4—Cd^vii^	100.934 (11)
Sb1^ii^—Eu1—Cd^ii^	85.892 (13)	Sb1—Eu4—Cd^vii^	134.792 (9)
Sb1^iii^—Eu1—Cd^ii^	143.242 (17)	Sb1^xv^—Eu4—Cd^vii^	45.207 (9)
Sb3^iv^—Eu1—Cd^ii^	46.924 (12)	Cd^xiv^—Eu4—Cd^vii^	106.514 (12)
Sb3^v^—Eu1—Cd^ii^	99.991 (17)	Cd^viii^—Eu4—Cd^vii^	73.486 (12)
Sb1^vi^—Eu1—Cd^ii^	45.530 (10)	Cd^xiii^—Eu4—Cd^vii^	180.000 (19)
Cd^vi^—Eu1—Cd^ii^	110.630 (13)	Sb2^xiii^—Eu4—Eu3^xxiv^	127.751 (9)
Eu1^xix^—Eu1—Cd^ii^	97.418 (10)	Sb2^vii^—Eu4—Eu3^xxiv^	52.249 (9)
Cd^iii^—Eu1—Cd^ii^	74.719 (14)	Sb2^xiv^—Eu4—Eu3^xxiv^	127.751 (9)
O^i^—Eu1—Eu2^iv^	37.31 (4)	Sb2^viii^—Eu4—Eu3^xxiv^	52.249 (9)
Sb1^ii^—Eu1—Eu2^iv^	55.018 (11)	Sb1—Eu4—Eu3^xxiv^	115.157 (11)
Sb1^iii^—Eu1—Eu2^iv^	104.009 (15)	Sb1^xv^—Eu4—Eu3^xxiv^	64.843 (11)
Sb3^iv^—Eu1—Eu2^iv^	67.923 (14)	Cd^xiv^—Eu4—Eu3^xxiv^	130.170 (8)
Sb3^v^—Eu1—Eu2^iv^	115.104 (14)	Cd^viii^—Eu4—Eu3^xxiv^	49.830 (8)
Sb1^vi^—Eu1—Eu2^iv^	145.294 (6)	Cd^xiii^—Eu4—Eu3^xxiv^	130.170 (8)
Cd^vi^—Eu1—Eu2^iv^	88.522 (13)	Cd^vii^—Eu4—Eu3^xxiv^	49.830 (8)
Eu1^xix^—Eu1—Eu2^iv^	62.674 (7)	Sb2^xiii^—Eu4—Eu3^xxv^	52.249 (9)
Cd^iii^—Eu1—Eu2^iv^	160.009 (14)	Sb2^vii^—Eu4—Eu3^xxv^	127.751 (9)
Cd^ii^—Eu1—Eu2^iv^	104.580 (11)	Sb2^xiv^—Eu4—Eu3^xxv^	52.249 (9)
O^i^—Eu1—Eu2^v^	37.31 (4)	Sb2^viii^—Eu4—Eu3^xxv^	127.751 (9)
Sb1^ii^—Eu1—Eu2^v^	104.009 (15)	Sb1—Eu4—Eu3^xxv^	64.843 (11)
Sb1^iii^—Eu1—Eu2^v^	55.018 (11)	Sb1^xv^—Eu4—Eu3^xxv^	115.157 (11)
Sb3^iv^—Eu1—Eu2^v^	115.104 (13)	Cd^xiv^—Eu4—Eu3^xxv^	49.830 (8)
Sb3^v^—Eu1—Eu2^v^	67.923 (14)	Cd^viii^—Eu4—Eu3^xxv^	130.170 (8)
Sb1^vi^—Eu1—Eu2^v^	145.294 (6)	Cd^xiii^—Eu4—Eu3^xxv^	49.830 (8)
Cd^vi^—Eu1—Eu2^v^	88.522 (12)	Cd^vii^—Eu4—Eu3^xxv^	130.170 (8)
Eu1^xix^—Eu1—Eu2^v^	62.674 (6)	Eu3^xxiv^—Eu4—Eu3^xxv^	180.000 (12)
Cd^iii^—Eu1—Eu2^v^	104.580 (11)	Cd^xiii^—Sb1—Cd^xiv^	111.92 (3)
Cd^ii^—Eu1—Eu2^v^	160.009 (14)	Cd^xiii^—Sb1—Eu1^xvi^	155.15 (2)
Eu2^iv^—Eu1—Eu2^v^	69.070 (12)	Cd^xiv^—Sb1—Eu1^xvi^	73.476 (13)
O^vii^—Eu2—O^viii^	126.7 (2)	Cd^xiii^—Sb1—Eu1^xvii^	73.476 (13)
O^vii^—Eu2—Sb1	82.09 (3)	Cd^xiv^—Sb1—Eu1^xvii^	155.15 (2)
O^viii^—Eu2—Sb1	82.09 (3)	Eu1^xvi^—Sb1—Eu1^xvii^	91.971 (17)
O^vii^—Eu2—Sb1^vi^	82.09 (3)	Cd^xiii^—Sb1—Eu4	79.416 (15)
O^viii^—Eu2—Sb1^vi^	82.09 (3)	Cd^xiv^—Sb1—Eu4	79.416 (15)
Sb1—Eu2—Sb1^vi^	144.21 (2)	Eu1^xvi^—Sb1—Eu4	125.146 (12)
O^vii^—Eu2—Sb2^viii^	151.15 (7)	Eu1^xvii^—Sb1—Eu4	125.146 (11)
O^viii^—Eu2—Sb2^viii^	72.66 (11)	Cd^xiii^—Sb1—Eu1^vi^	77.026 (16)
Sb1—Eu2—Sb2^viii^	79.950 (11)	Cd^xiv^—Sb1—Eu1^vi^	77.026 (16)
Sb1^vi^—Eu2—Sb2^viii^	124.796 (11)	Eu1^xvi^—Sb1—Eu1^vi^	80.908 (14)
O^vii^—Eu2—Sb2^xx^	151.15 (7)	Eu1^xvii^—Sb1—Eu1^vi^	80.908 (14)
O^viii^—Eu2—Sb2^xx^	72.66 (11)	Eu4—Sb1—Eu1^vi^	137.202 (18)
Sb1—Eu2—Sb2^xx^	124.795 (11)	Cd^xiii^—Sb1—Eu2	118.413 (15)
Sb1^vi^—Eu2—Sb2^xx^	79.951 (11)	Cd^xiv^—Sb1—Eu2	118.413 (15)
Sb2^viii^—Eu2—Sb2^xx^	46.098 (17)	Eu1^xvi^—Sb1—Eu2	75.300 (13)
O^vii^—Eu2—Sb2^xxi^	72.66 (11)	Eu1^xvii^—Sb1—Eu2	75.300 (13)
O^viii^—Eu2—Sb2^xxi^	151.15 (7)	Eu4—Sb1—Eu2	77.364 (14)
Sb1—Eu2—Sb2^xxi^	124.795 (11)	Eu1^vi^—Sb1—Eu2	145.435 (19)
Sb1^vi^—Eu2—Sb2^xxi^	79.951 (11)	Sb2^vi^—Sb2—Cd	109.800 (15)
Sb2^viii^—Eu2—Sb2^xxi^	99.72 (2)	Sb2^vi^—Sb2—Eu4^ix^	133.614 (8)
Sb2^xx^—Eu2—Sb2^xxi^	82.082 (16)	Cd—Sb2—Eu4^ix^	78.379 (13)
O^vii^—Eu2—Sb2^vii^	72.66 (11)	Sb2^vi^—Sb2—Eu4^x^	133.614 (8)
O^viii^—Eu2—Sb2^vii^	151.15 (7)	Cd—Sb2—Eu4^x^	78.379 (13)
Sb1—Eu2—Sb2^vii^	79.950 (11)	Eu4^ix^—Sb2—Eu4^x^	92.637 (15)
Sb1^vi^—Eu2—Sb2^vii^	124.796 (11)	Sb2^vi^—Sb2—Eu3^viii^	65.699 (9)
Sb2^viii^—Eu2—Sb2^vii^	82.082 (17)	Cd—Sb2—Eu3^viii^	65.122 (14)
Sb2^xx^—Eu2—Sb2^vii^	99.72 (2)	Eu4^ix^—Sb2—Eu3^viii^	78.764 (8)
Sb2^xxi^—Eu2—Sb2^vii^	46.098 (17)	Eu4^x^—Sb2—Eu3^viii^	143.452 (18)
O^vii^—Eu2—Eu1^iv^	35.58 (8)	Sb2^vi^—Sb2—Eu3^vii^	65.699 (9)
O^viii^—Eu2—Eu1^iv^	101.55 (11)	Cd—Sb2—Eu3^vii^	65.122 (14)
Sb1—Eu2—Eu1^iv^	103.103 (14)	Eu4^ix^—Sb2—Eu3^vii^	143.452 (18)
Sb1^vi^—Eu2—Eu1^iv^	49.682 (9)	Eu4^x^—Sb2—Eu3^vii^	78.764 (8)
Sb2^viii^—Eu2—Eu1^iv^	173.179 (9)	Eu3^viii^—Sb2—Eu3^vii^	87.282 (17)
Sb2^xx^—Eu2—Eu1^iv^	129.306 (11)	Sb2^vi^—Sb2—Eu2^x^	66.952 (9)
Sb2^xxi^—Eu2—Eu1^iv^	83.628 (11)	Cd—Sb2—Eu2^x^	137.661 (10)
Sb2^vii^—Eu2—Eu1^iv^	104.387 (9)	Eu4^ix^—Sb2—Eu2^x^	136.289 (17)
O^vii^—Eu2—Eu1^xvi^	35.58 (8)	Eu4^x^—Sb2—Eu2^x^	76.887 (8)
O^viii^—Eu2—Eu1^xvi^	101.55 (11)	Eu3^viii^—Sb2—Eu2^x^	132.515 (16)
Sb1—Eu2—Eu1^xvi^	49.683 (9)	Eu3^vii^—Sb2—Eu2^x^	76.673 (13)
Sb1^vi^—Eu2—Eu1^xvi^	103.102 (15)	Sb2^vi^—Sb2—Eu2^ix^	66.952 (8)
Sb2^viii^—Eu2—Eu1^xvi^	129.306 (11)	Cd—Sb2—Eu2^ix^	137.661 (10)
Sb2^xx^—Eu2—Eu1^xvi^	173.179 (9)	Eu4^ix^—Sb2—Eu2^ix^	76.887 (7)
Sb2^xxi^—Eu2—Eu1^xvi^	104.387 (10)	Eu4^x^—Sb2—Eu2^ix^	136.289 (17)
Sb2^vii^—Eu2—Eu1^xvi^	83.628 (11)	Eu3^viii^—Sb2—Eu2^ix^	76.673 (14)
Eu1^iv^—Eu2—Eu1^xvi^	54.653 (13)	Eu3^vii^—Sb2—Eu2^ix^	132.515 (16)
O^vii^—Eu2—Eu1^v^	101.55 (11)	Eu2^x^—Sb2—Eu2^ix^	82.083 (17)
O^viii^—Eu2—Eu1^v^	35.58 (8)	Cd—Sb3—Cd^vi^	111.30 (3)
Sb1—Eu2—Eu1^v^	103.103 (15)	Cd—Sb3—Eu1^xvii^	76.384 (11)
Sb1^vi^—Eu2—Eu1^v^	49.682 (9)	Cd^vi^—Sb3—Eu1^xvii^	135.084 (7)
Sb2^viii^—Eu2—Eu1^v^	104.387 (9)	Cd—Sb3—Eu1^iv^	135.084 (7)
Sb2^xx^—Eu2—Eu1^v^	83.628 (11)	Cd^vi^—Sb3—Eu1^iv^	76.384 (11)
Sb2^xxi^—Eu2—Eu1^v^	129.306 (11)	Eu1^xvii^—Sb3—Eu1^iv^	130.47 (3)
Sb2^vii^—Eu2—Eu1^v^	173.179 (9)	Cd—Sb3—Eu1^v^	135.084 (7)
Eu1^iv^—Eu2—Eu1^v^	69.069 (12)	Cd^vi^—Sb3—Eu1^v^	76.384 (12)
Eu1^xvi^—Eu2—Eu1^v^	93.682 (16)	Eu1^xvii^—Sb3—Eu1^v^	69.701 (15)
O^vii^—Eu2—Eu1^xvii^	101.55 (11)	Eu1^iv^—Sb3—Eu1^v^	89.770 (15)
O^viii^—Eu2—Eu1^xvii^	35.58 (8)	Cd—Sb3—Eu1^xvi^	76.384 (11)
Sb1—Eu2—Eu1^xvii^	49.683 (9)	Cd^vi^—Sb3—Eu1^xvi^	135.084 (7)
Sb1^vi^—Eu2—Eu1^xvii^	103.102 (14)	Eu1^xvii^—Sb3—Eu1^xvi^	89.770 (15)
Sb2^viii^—Eu2—Eu1^xvii^	83.628 (11)	Eu1^iv^—Sb3—Eu1^xvi^	69.701 (15)
Sb2^xx^—Eu2—Eu1^xvii^	104.387 (9)	Eu1^v^—Sb3—Eu1^xvi^	130.47 (3)
Sb2^xxi^—Eu2—Eu1^xvii^	173.179 (9)	Cd—Sb3—Eu3^vii^	66.237 (13)
Sb2^vii^—Eu2—Eu1^xvii^	129.306 (11)	Cd^vi^—Sb3—Eu3^vii^	66.236 (13)
Eu1^iv^—Eu2—Eu1^xvii^	93.682 (16)	Eu1^xvii^—Sb3—Eu3^vii^	142.479 (13)
Eu1^xvi^—Eu2—Eu1^xvii^	69.069 (12)	Eu1^iv^—Sb3—Eu3^vii^	78.764 (10)
Eu1^v^—Eu2—Eu1^xvii^	54.653 (13)	Eu1^v^—Sb3—Eu3^vii^	142.479 (13)
Sb3^ix^—Eu3—Sb3^x^	88.85 (2)	Eu1^xvi^—Sb3—Eu3^vii^	78.764 (10)
Sb3^ix^—Eu3—Sb2^xi^	155.259 (10)	Cd—Sb3—Eu3^viii^	66.237 (13)
Sb3^x^—Eu3—Sb2^xi^	86.675 (12)	Cd^vi^—Sb3—Eu3^viii^	66.236 (14)
Sb3^ix^—Eu3—Sb2^x^	155.259 (10)	Eu1^xvii^—Sb3—Eu3^viii^	78.764 (10)
Sb3^x^—Eu3—Sb2^x^	86.675 (12)	Eu1^iv^—Sb3—Eu3^viii^	142.479 (13)
Sb2^xi^—Eu3—Sb2^x^	48.601 (18)	Eu1^v^—Sb3—Eu3^viii^	78.764 (10)
Sb3^ix^—Eu3—Sb2^ix^	86.675 (12)	Eu1^xvi^—Sb3—Eu3^viii^	142.479 (13)
Sb3^x^—Eu3—Sb2^ix^	155.259 (10)	Eu3^vii^—Sb3—Eu3^viii^	88.86 (2)
Sb2^xi^—Eu3—Sb2^ix^	106.94 (2)	Sb1^xiii^—Cd—Sb1^xiv^	111.92 (3)
Sb2^x^—Eu3—Sb2^ix^	87.283 (17)	Sb1^xiii^—Cd—Sb3	111.885 (17)
Sb3^ix^—Eu3—Sb2^xii^	86.675 (12)	Sb1^xiv^—Cd—Sb3	111.885 (17)
Sb3^x^—Eu3—Sb2^xii^	155.259 (10)	Sb1^xiii^—Cd—Sb2	108.095 (17)
Sb2^xi^—Eu3—Sb2^xii^	87.283 (17)	Sb1^xiv^—Cd—Sb2	108.095 (17)
Sb2^x^—Eu3—Sb2^xii^	106.94 (2)	Sb3—Cd—Sb2	104.55 (2)
Sb2^ix^—Eu3—Sb2^xii^	48.601 (18)	Sb1^xiii^—Cd—Eu3^vii^	166.858 (18)
Sb3^ix^—Eu3—Cd^ix^	50.649 (11)	Sb1^xiv^—Cd—Eu3^vii^	80.981 (12)
Sb3^x^—Eu3—Cd^ix^	108.724 (16)	Sb3—Cd—Eu3^vii^	63.115 (14)
Sb2^xi^—Eu3—Cd^ix^	152.666 (19)	Sb2—Cd—Eu3^vii^	63.735 (14)
Sb2^x^—Eu3—Cd^ix^	108.315 (13)	Sb1^xiii^—Cd—Eu3^viii^	80.981 (12)
Sb2^ix^—Eu3—Cd^ix^	51.142 (13)	Sb1^xiv^—Cd—Eu3^viii^	166.858 (18)
Sb2^xii^—Eu3—Cd^ix^	86.946 (12)	Sb3—Cd—Eu3^viii^	63.115 (14)
Sb3^ix^—Eu3—Cd^xi^	108.724 (17)	Sb2—Cd—Eu3^viii^	63.735 (14)
Sb3^x^—Eu3—Cd^xi^	50.649 (11)	Eu3^vii^—Cd—Eu3^viii^	86.030 (15)
Sb2^xi^—Eu3—Cd^xi^	51.142 (13)	Sb1^xiii^—Cd—Eu1^vi^	58.674 (13)
Sb2^x^—Eu3—Cd^xi^	86.946 (12)	Sb1^xiv^—Cd—Eu1^vi^	58.674 (13)
Sb2^ix^—Eu3—Cd^xi^	152.666 (19)	Sb3—Cd—Eu1^vi^	109.99 (2)
Sb2^xii^—Eu3—Cd^xi^	108.315 (14)	Sb2—Cd—Eu1^vi^	145.46 (2)
Cd^ix^—Eu3—Cd^xi^	154.68 (3)	Eu3^vii^—Cd—Eu1^vi^	133.990 (11)
Sb3^ix^—Eu3—Cd^xii^	50.649 (11)	Eu3^viii^—Cd—Eu1^vi^	133.990 (11)
Sb3^x^—Eu3—Cd^xii^	108.724 (17)	Sb1^xiii^—Cd—Eu1^xvii^	57.443 (13)
Sb2^xi^—Eu3—Cd^xii^	108.315 (13)	Sb1^xiv^—Cd—Eu1^xvii^	117.87 (2)
Sb2^x^—Eu3—Cd^xii^	152.666 (19)	Sb3—Cd—Eu1^xvii^	56.693 (13)
Sb2^ix^—Eu3—Cd^xii^	86.946 (12)	Sb2—Cd—Eu1^xvii^	133.962 (13)
Sb2^xii^—Eu3—Cd^xii^	51.142 (13)	Eu3^vii^—Cd—Eu1^xvii^	119.724 (18)
Cd^ix^—Eu3—Cd^xii^	88.461 (16)	Eu3^viii^—Cd—Eu1^xvii^	70.603 (12)
Cd^xi^—Eu3—Cd^xii^	86.030 (15)	Eu1^vi^—Cd—Eu1^xvii^	69.370 (13)
Sb3^ix^—Eu3—Cd^x^	108.724 (16)	Sb1^xiii^—Cd—Eu1^xvi^	117.87 (2)
Sb3^x^—Eu3—Cd^x^	50.649 (11)	Sb1^xiv^—Cd—Eu1^xvi^	57.443 (13)
Sb2^xi^—Eu3—Cd^x^	86.946 (12)	Sb3—Cd—Eu1^xvi^	56.693 (13)
Sb2^x^—Eu3—Cd^x^	51.142 (13)	Sb2—Cd—Eu1^xvi^	133.962 (13)
Sb2^ix^—Eu3—Cd^x^	108.315 (13)	Eu3^vii^—Cd—Eu1^xvi^	70.603 (12)
Sb2^xii^—Eu3—Cd^x^	152.666 (19)	Eu3^viii^—Cd—Eu1^xvi^	119.724 (18)
Cd^ix^—Eu3—Cd^x^	86.030 (15)	Eu1^vi^—Cd—Eu1^xvi^	69.370 (13)
Cd^xi^—Eu3—Cd^x^	88.461 (16)	Eu1^xvii^—Cd—Eu1^xvi^	74.720 (15)
Cd^xii^—Eu3—Cd^x^	154.68 (3)	Sb1^xiii^—Cd—Eu4^x^	115.04 (2)
Sb3^ix^—Eu3—Eu4^xxii^	111.194 (5)	Sb1^xiv^—Cd—Eu4^x^	55.378 (13)
Sb3^x^—Eu3—Eu4^xxii^	111.194 (5)	Sb3—Cd—Eu4^x^	132.546 (13)
Sb2^xi^—Eu3—Eu4^xxii^	48.986 (9)	Sb2—Cd—Eu4^x^	54.120 (12)
Sb2^x^—Eu3—Eu4^xxii^	93.069 (14)	Eu3^vii^—Cd—Eu4^x^	69.550 (10)
Sb2^ix^—Eu3—Eu4^xxii^	93.069 (14)	Eu3^viii^—Cd—Eu4^x^	117.827 (18)
Sb2^xii^—Eu3—Eu4^xxii^	48.986 (8)	Eu1^vi^—Cd—Eu4^x^	99.961 (13)
Cd^ix^—Eu3—Eu4^xxii^	135.440 (10)	Eu1^xvii^—Cd—Eu4^x^	168.941 (17)
Cd^xi^—Eu3—Eu4^xxii^	60.620 (10)	Eu1^xvi^—Cd—Eu4^x^	104.797 (9)
Cd^xii^—Eu3—Eu4^xxii^	60.620 (10)	Sb1^xiii^—Cd—Eu4^ix^	55.378 (13)
Cd^x^—Eu3—Eu4^xxii^	135.440 (10)	Sb1^xiv^—Cd—Eu4^ix^	115.04 (2)
Sb3^ix^—Eu3—Eu4^xxiii^	111.194 (5)	Sb3—Cd—Eu4^ix^	132.546 (13)
Sb3^x^—Eu3—Eu4^xxiii^	111.194 (5)	Sb2—Cd—Eu4^ix^	54.120 (12)
Sb2^xi^—Eu3—Eu4^xxiii^	93.069 (14)	Eu3^vii^—Cd—Eu4^ix^	117.827 (18)
Sb2^x^—Eu3—Eu4^xxiii^	48.986 (8)	Eu3^viii^—Cd—Eu4^ix^	69.550 (10)
Sb2^ix^—Eu3—Eu4^xxiii^	48.986 (8)	Eu1^vi^—Cd—Eu4^ix^	99.961 (13)
Sb2^xii^—Eu3—Eu4^xxiii^	93.069 (14)	Eu1^xvii^—Cd—Eu4^ix^	104.797 (9)
Cd^ix^—Eu3—Eu4^xxiii^	60.620 (10)	Eu1^xvi^—Cd—Eu4^ix^	168.941 (17)
Cd^xi^—Eu3—Eu4^xxiii^	135.440 (10)	Eu4^x^—Cd—Eu4^ix^	73.485 (12)
Cd^xii^—Eu3—Eu4^xxiii^	135.440 (10)	Eu1^i^—O—Eu1^xviii^	97.9 (2)
Cd^x^—Eu3—Eu4^xxiii^	60.620 (10)	Eu1^i^—O—Eu2^ix^	107.12 (4)
Eu4^xxii^—Eu3—Eu4^xxiii^	119.173 (14)	Eu1^xviii^—O—Eu2^ix^	107.12 (4)
Sb2^xiii^—Eu4—Sb2^vii^	180.00 (2)	Eu1^i^—O—Eu2^x^	107.12 (4)
Sb2^xiii^—Eu4—Sb2^xiv^	92.638 (15)	Eu1^xviii^—O—Eu2^x^	107.12 (4)
Sb2^vii^—Eu4—Sb2^xiv^	87.362 (15)	Eu2^ix^—O—Eu2^x^	126.7 (2)

Symmetry codes: (i) −*x*, −*y*+1, −*z*+1; (ii) −*x*+1/2, −*y*+1/2, *z*+1/2; (iii) −*x*−1/2, −*y*+1/2, *z*+1/2; (iv) −*x*+1/2, −*y*+1/2, −*z*+1; (v) −*x*−1/2, −*y*+1/2, −*z*+1; (vi) *x*, *y*, −*z*+1/2; (vii) *x*+1/2, *y*−1/2, *z*; (viii) *x*−1/2, *y*−1/2, *z*; (ix) *x*−1/2, *y*+1/2, *z*; (x) *x*+1/2, *y*+1/2, *z*; (xi) *x*+1/2, *y*+1/2, −*z*+1/2; (xii) *x*−1/2, *y*+1/2, −*z*+1/2; (xiii) −*x*−1/2, −*y*+1/2, −*z*; (xiv) −*x*+1/2, −*y*+1/2, −*z*; (xv) −*x*, −*y*, −*z*; (xvi) −*x*+1/2, −*y*+1/2, *z*−1/2; (xvii) −*x*−1/2, −*y*+1/2, *z*−1/2; (xviii) −*x*, −*y*+1, *z*−1/2; (xix) *x*, *y*, −*z*+3/2; (xx) *x*−1/2, *y*−1/2, −*z*+1/2; (xxi) *x*+1/2, *y*−1/2, −*z*+1/2; (xxii) −*x*, −*y*+1, *z*+1/2; (xxiii) *x*, *y*+1, *z*; (xxiv) *x*, *y*−1, *z*; (xxv) −*x*, −*y*+1, −*z*.
